# i-Tree cool river: An open source, freeware tool to simulate river water temperature coupled with HEC-RAS

**DOI:** 10.1016/j.mex.2020.100808

**Published:** 2020-02-20

**Authors:** Reza Abdi, Theodore Endreny, David Nowak

**Affiliations:** a Department of Civil and Environmental Engineering, Colorado School of Mines, Golden, CO 80401, USA; b Department of Environmental Resources Engineering, State University of New York, College of Environmental Science and Forestry, Syracuse, NY 13210, USA; c USDA Forest Service, SUNY-ESF, Northern Research Station, 5 Moon Library, Syracuse, NY 13210, USA

**Keywords:** Thermal pollution, Aquatic thermal habitat, Mechanistic model, Energy balance

## Abstract

This method paper explains the i-Tree Cool River model algorithms for simulating the response of river water temperature to urban greening. The model captures the warming and cooling impacts of urban development and restoration through a water and energy budget. The water budget includes river inflows from urban storm sewers and reservoirs, and the associated water temperatures. The energy budget adjusts radiation fluxes due to riparian shading and evapotranspiration, and propagates temperature downstream. Restorative cooling of the river can be simulated through algorithms for cool groundwater, either as direct inflows or by river water replacement called hyporheic exchange. Novel features in the model include diurnal variation in riparian shading, use of the Army Corps of Engineers HEC-RAS model predicted river depths and velocities, and periodic boundary conditions to rapidly extend restoration scenarios.

• Freely available code in C++ for Visual Studio, with a detailed manual and sample inputs and outputs at http://www.itreetools.org/research_suite/coolriver.
• Useful for simulating river warming or cooling due to urban development or greening.
• Well documented source code compatible with requirements of other modeling groups.

## Graphical abstract

**Figure:**
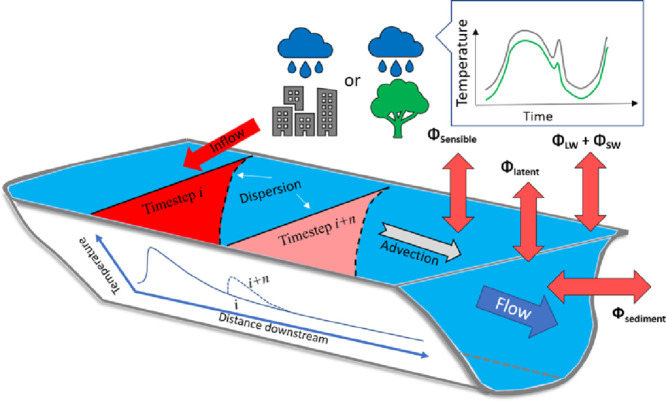

**Table:** Specifications table

Subject Area	Environmental Science
More specific subject area	*[Describe narrower subject area]* River water quality
Method name	*[Please specify a name of the method that you customized]* i-Tree Cool River
Name and reference of original method	*[If applicable, include full bibliographic details of the main reference(s) describing the original method from which the new method was derived.]* • Martin, J.L.; McCutcheon, S.C. Hydrodynamics and Transport for Water Quality Modeling; Lewis Publishers: New York, NY, USA, 1999. • Boyd, M.; Kasper, B. Analytical Methods for Dynamic Open Channel Heat and Mass Transfer: Methodology for Heat Source Model Version 7.0; Watershed Sciences Inc.: Portland, OR, USA, 2003. • Chen, Y.D.; Carsel, R.F.; McCutcheon, S.C.; Nutter, W.L. Stream temperature simulation of forested riparian areas: I. Watershed-Scale model development. J. Environ. Eng. 1998.
Resource availability	http://www.itreetools.org/research_suite/coolriver

## Method details

### Model description

The i-Tree Cool River model's main equation for simulating the river temperature is based on an advection-dispersion relationship which includes the impacts of the lateral inflows and heat fluxes affecting the simulated river temperature [1], [2], [3]:

(1) $$\frac{\partial T_{w}}{\partial t}=-U\frac{\partial T_{w}}{\partial x}+D_{L}\frac{\partial^{2}T_{w}}{\partial x^{2}}+R_{h}+R_{i}$$

where *T_w_* is the cross-sectional averaged river temperature (°C), *t* is time in the simulation (s), *U* is the reach average flow velocity (m/s), *∂x* is river cross-sectional intervals (m), *D_L_* is the dispersion coefficient (m^2^/s), *R_h_* is the heat flux reaction term, and *R_i_* is the reaction term of the external inflows. Combining the reaction term, *R_i_*, with the first (advection) and second (dispersion) terms in the right-hand side in the Eq. (1), altogether define the mass transfer [2,3,4]. The *R_h_* and *R_i_* are defined as:

(2) $$R_{h}=\frac{\Phi_{net}}{\rho C_{p}y}$$

(3) $$R_{i}=\frac{Q_{W}T_{W}+Q_{GW}T_{GW}+Q_{Hyp}T_{Hyp}+Q_{SS}T_{SS}}{Q_{i}+Q_{GW}+Q_{Hyp}+Q_{SS}}-T_{W,t-1}$$

where Φ_*net*_ is the net thermal energy (W/m^2^), *ρ* is the water density as 1000 (kg/m^3^), *C_p_* is the specific heat capacity of water which is defined as 4182 (J/kg °C), *y* is the average water column depth (m), *Q* is the discharge (m^3^/s), *T* is water temperature (°C), given that the subscripts *W* represents the river, *GW* represents the groundwater, *Hyp* represents the hyporheic exchange, and *SS* represents the stormwater inflow. In the Eq. (3) the *t-1* refers to the prior time step. For surface inflows in Eq. (3) represented by the subscript *SS*, the users can include an unlimited number of surface inflows for each cross-section.

The i-Tree Cool River Model uses inputs of river discharge to solve for the advection and dispersion terms in Eq. (1) as well as solve for the inflow reaction term, *R_i_* in Eqs. (1) and (3). In unsteady conditions, such as during a storm event, the model determines river velocity and dispersion using the one-dimensional St. Venant equation, which is solved numerically using the finite difference method given in Eqs. (3)–(25) to (3)–(29) by Boyd and Kasper [2]. This St. Venant finite difference method uses the Manning equation to relate velocity with river water depth, wetted perimeter, and cross-sectional area. The Manning equation operates in trapezoidal, triangular, or square channels with prescribed width, roughness, and side slope. The i-Tree Cool River model uses a version of the Manning equation provided by Boyd and Kasper [2] in Eq. (3)–(11) [3]. The Newton-Raphson root-finding iterative method is used to solve the Manning equation and determine the adjusted wetted depth, hydraulic radius, wetted perimeter, cross-sectional area, and bottom width [2]. The i-Tree Cool River model uses the estimated velocity with the MacCormick method to determine the rate at which river water temperature travels between cross-sections, using Eqs. (2-119) to (2-122) from Boyd and Kasper [2]. The St. Venant finite difference method requires compliance with Courant and frictional stability conditions for each node every timestep, using Eqs. (3-30) and (3-31) of Boyd and Kasper [2]. In steady-state conditions, the model can determine velocity and dispersion using the St. Venant method, as done by Boyd and Kasper [2], or the user can select the Crank-Nicolson numerical method [5,6] to solve a coupled set of velocity and temperature equations [3].

Inflows are composed of surface and subsurface sources. The surface inflow terms, *Q_ss_* and *T_ss_* of Eq. (3) are input as a time series of flow rate (m^3^/s) and temperature (°C), respectively, for any node receiving storm sewer, tributary, or other surface inflows. The flow and temperature values are either provided through measured observation or through estimation. The subsurface terms for groundwater inflow, *Q_GW_* and *T_GW_* of Eq. (3) are input as a time series of groundwater flow rate (m^3^/s) and temperature ( °C) for each node and can be based on observation or estimation. The groundwater temperature is set to a constant value based on a function of annual average air temperature warming slightly in the summertime and cooling slightly in the wintertime as suggested in the literature [6]. Groundwater inflow is determined from observation, or based on measuring baseflow at the upstream (station at the 0 m) and downstream (station at the end of the reach) sections during dry weather_,_ and computing the inflow rate per unit length of the reach. The subsurface hyporheic flow rate (m^3^/s), *Q_Hyp,_* and hyporheic flow temperature (°C), *T_Hyp_* terms of Eq. (3) for each node can be based on observation or estimation. Similar to groundwater flow, the hyporheic temperature is set to the constant value. The hyporheic inflow is calculated in the model based on the Darcy Law [7] as

(4) $$Q_{Hyp}=A_{S}K_{S}\frac{dh_{D}}{dx}$$

where *A_S_* is cross-sectional across seepage face (m^2^), *K_S_* is dominant substrate hydraulic conductivity (m/s), *h_D_* is hydraulic head for Darcy calculation (m), and *x* is the model distance step (m).

The net exchange of thermal energy in Eq. (2) is defined as a combination of energy fluxes as in Boyd and Kasper [2] as

(5) $$\Phi_{net}=\Phi_{longwave}+\Phi_{shortwave}+\Phi_{latent}+\Phi_{sensible}+\Phi_{sediment}$$

where theΦ is the heat flux (W/m^2^), and subscripts *_net_* is the net heat flux at the water surface, *_longwave_* is the longwave radiation flux at the water surface, *_shortwave_* is the shortwave radiation at the water surface, *_latent_* is the latent heat flux from evaporation, *_sensible_* is the sensible heat flux representing the convective thermal flux from the water surface, and *_sediment_* is the bed sediment heat flux representing conduction forcing at the water column interface [3].

### Longwave radiation flux

**Fig. 1:**
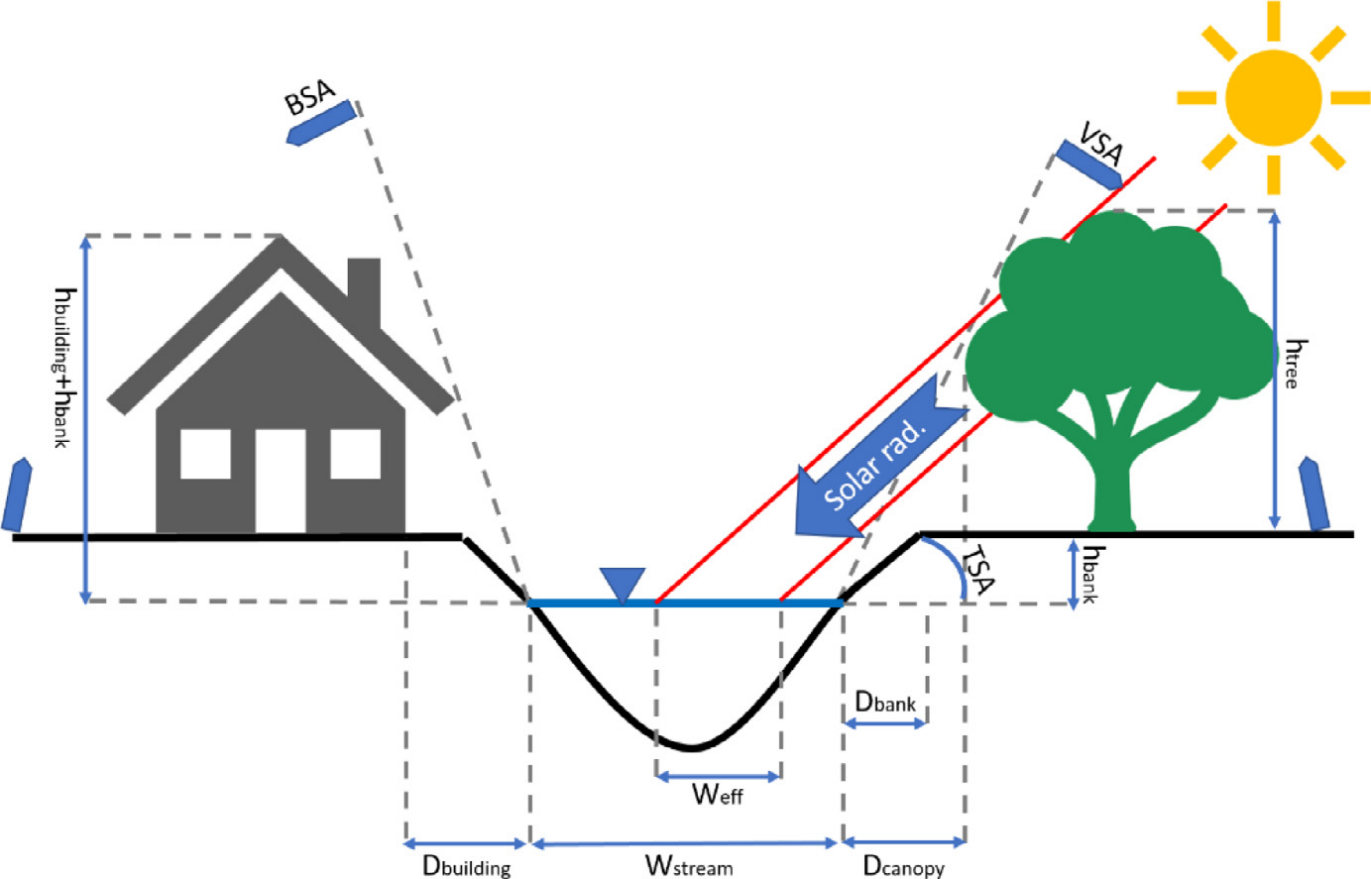
Schematic of a river cross-section where the BSA, TSA, and VSA terms are shading angles due to building, topography, and vegetation respectively. h_building_, h_tree_, and h_bank_ are building, vegetation, and bank heights respectively and the D_building_, D_canopy_, and D_bank_ terms are the distances of the building, canopy, and bank respectively to the edge of the water.

The longwave radiation flux in Eq. (5) includes three terms determining positive downward fluxes from the atmosphere and land cover over the water surface, and a negative upward flux from the waterbody to the air [2,3]

(6) $$\Phi_{longwave}=\Phi_{longwave}^{atmospheric}+\Phi_{longwave}^{land\text{cov}er}+\Phi_{longwave}^{back}$$

where $\Phi_{longwave}^{atmospheric}$ is the atmospheric flux (W/m^2^), $\Phi_{longwave}^{land\text{cov}er}$ is the land cover flux (W/m^2^), and $\Phi_{longwave}^{back}$ is the back-to-air flux (W/m^2^). Atmospheric longwave radiation is a function of air temperature and exposure from the river surface to the atmosphere, called the view-to-sky factor (*f*), calculated using Boyd et al. [27]

(7) $$\Phi_{longwave}^{atmospheric}=0.96\varepsilon_{atm}\sigma {\left(T_{air}+273.2\right)}^{4}\min\left(f_{1},f_{2},f_{3}\right)$$

where *T_air_* is air temperature ( °C), the ɛ_*atm*_ is the emissivity of the atmosphere (0 to 1), *σ* is the Stefan-Boltzmann constant (5.6696 × 10^−8^, W/m^2^K^4^), and min(*f*_1_*, f*_2_*, f*_3_) is the minimum of the three view-to-sky factors (0 to 1), including the *f*_1_ as the building effects, the *f*_2_ as the vegetation effects, and the *f*_3_ as the topographic effects (Fig. 1). The emissivity of the atmosphere ɛ_*atm*_ is calculated using [8]

(8) $$\varepsilon_{atm}=1.72{\left(\frac{0.1e_{a}}{T_{air}+273.2}\right)}^{\frac{1}{7}}\left(1+0.22{C_{L}}^{2}\right)$$

where *e_a_* is the actual vapor pressure (mbar) and *C_L_* is the cloudiness factor, which ranges from 0 for a clear sky to 1 for full cloud cover and can be found from the weather station data or can be estimated from comparison between the solar radiation in the edge of the atmosphere and on the ground [9].

The view-to-sky factors with a value of 1 indicate a full unobstructed sky view and with 0 indicates a blocked condition for the solar radiation [2,10]. The general sky-view-factor formula for *f_i_* is computed for all the cross-sections with the determined interval [11]

(9) $$f_{i}=1-\frac{2}{\pi }SA_{i}$$

where *i* indicates the object at that cross-section (1 for the building, 2 for the vegetation, and 3 for the topography), and *SA* is the shade angle (radians), computed as and *h_c_* is the combined height of the objects above the water (e.g., if a tree is set on a hill, *h_c_* = *h_tree_* + *h_bank_*), and max (*D_i_*) is the maximum distance from all objects at that cross-section to the edge of the water.

(10) $$SA_{i}=\tan^{-1}\left(\frac{h_{c}}{\max(D_{i,1-3})}\right)$$

The landcover longwave radiation in Eq. (6) also uses the view-to-sky factor parameters as in Eq. (7). The land cover radiation flux represents the land cover effect, e.g., vegetation cover's influence on water temperature in the form of longwave radiation. The model by default sets land cover temperature equal to atmospheric temperature, following the approach of Boyd and Kasper [2]

(11) $$\Phi_{longwave}^{land\text{cov}er}=0.96\left(1-\min\left(f_{1},f_{2},f_{3}\right)\right)0.96\sigma {\left(T_{air}+273.2\right)}^{4}$$

The waterbody to air radiation term in Eq. (5) is a function of water temperature, representing heat flux emitted from the water surface, following the approach of Boyd and Kasper [2]

(12) $$\Phi_{longwave}^{back}=-0.96\sigma {\left(T_{w}+273.2\right)}^{4}$$

where the *T_W_* is the river temperature ( °C).

#### Shortwave radiation (first method)

The model provides two methods for calculating shortwave radiation. The first method calculates the total shortwave radiation in Eq. (5) is a function of the incoming solar radiation [12], based on the albedo and a shading factor, which is based on the riparian vegetation condition along the river corridor [6,11]

(13) $$\Phi_{shortwave}=S_{in}\left(1-a\right)\left(1-SF\right)$$

where S_in_ is incoming shortwave radiation, as the sum of direct and diffuse shortwave radiations, *a* is the albedo (ranging from 0 to 1), and *SF* is the estimated shading factor (0 to 1, with 1 for complete shade).

#### Shortwave radiation (second method)

The second method for evaluating the shortwave radiation combines the adjusted direct and diffuse shortwave radiation and uses sky view factors (discussed in section 1.1) and shading width in place of a shading factor [3,15]

(14) $$\Phi_{shortwave}=\Phi_{shortwave}^{{}^{\prime \prime }direct}+\Phi_{shortwave}^{{}^{\prime }diffuse}$$

The view-to-sky factor is applied to compute the topographic shading effect on diffuse solar radiation ($S_{shortwave}^{diffuse}$) [11,15]

(15) $$\Phi_{shortwave}^{{}^{\prime }diffuse}=S_{shortwave}^{diffuse}\left(1-a\right)\min\left(f_{1},f_{2},f_{3}\right)$$

Direct shortwave radiation is computed using a two-step adjustment process, accounting for the width of shade across the river surface, and the river slope and aspect, as well as the slope, aspect, solar azimuth, as well as solar altitude [13]

(16) $$\Phi_{shortwave}^{{}^{\prime \prime }direct}=\Phi_{shortwave}^{{}^{\prime }direct}\left(1-\frac{{W^{\prime }}_{ef}}{W_{river}}\right)$$

where *W’_eff_* is the width of the effective shading and *W_river_* is the river section wetted width. The width of the effective shading and river section wetted width are explained in the next paragraph. The adjusted shortwave radiation (${\Phi^{\prime }}_{shortwave}^{direct}$) is calculated as [13]

(17) $$\Phi_{shortwave}^{{}^{\prime }direct}=S_{shortwave}^{direct}\left(1-a\right)\left[\sin\alpha \cos\varphi \cos(\beta -\theta_{\text{sun}})+\cos\alpha \sin\varphi \right]$$

where $S_{shortwave}^{direct}$ is the incoming direct shortwave radiation can be imported to the model as an input file, *α* is the longitudinal water surface slope (radians), *β* is the aspect with considering the 0 as the true north (radians), *θ_sun_* is solar azimuth angle (radians), indicating the angle of the position of the sun relative to true north, and *φ* is solar altitude (radians). The second method for calculating shortwave radiation can reduce to the first method, in cases of full shade and full sun. For the case of full sun, the shade angle, *SA* = 0 and *f_i_* = 1, resulting in $\Phi_{shortwave}^{{}^{\prime }diffuse}$= $S_{shortwave}^{diffuse}\left(1-a\right)$ for Eq. (14), and the complementary term for the $\Phi_{shortwave}^{{}^{\prime \prime }direct}$, becomes $\Phi_{shortwave}^{{}^{\prime }direct}$= $S_{shortwave}^{direct}\left(1-a\right)$ when φ = *π/2* in Eq. (17) and $W_{eff}^{\prime }$= 0 in Eq. (16). For the case of full shade, the corollary occurs, with *SA* = 1 and *f_i_* = 0, and $W_{eff}^{\prime }$= *W_river_* in Eq. (15), resulting in no solar radiation on the river.

The total width for the shading in the river cross-section, *W_shade_*, caused by near river objects, is calculated at each time step as a function of solar azimuth, altitude, and river azimuth (*θ_river_*), in addition to object height at each node [13]

(18) $$W_{shade}=\left(h_{i}\right)\left|\frac{\sin(\theta_{sun}-\theta_{river})}{\tan\varphi }\right|$$

where the *h_i_* is the combined height of the topography and building or vegetation bordering the river (*i* = 3) depending on the cross-sectional condition. When building and vegetation are present, the object is selected based on which has the largest shade angle *SA* from Eq. (10). The river width and distance from river to the shading object are compared with *W_shade_* to determine the distances across the river cross-section surface covered in shade and to determine the width of river effectively shaded (*W_eff_*) and the width of the river directly under an overhanging object, *W_overhang_* such as tree canopy [11]. The model estimates the tree canopy width protruding from the tree trunk midpoint as 10% of the tree height base on the suggestion by Chen et al., [11]. The overhang is computed for either left or right banks as [11]

(19) $$W_{overhang}=\left\{ \begin{array}{ccc} \left(0.1h_{tree}-D_{canopy}\right)\rho_{veg} & \text{if} & \left[\left(0.1h_{tree}-D_{canopy}\right)<W_{stream}\right]\\ W_{stream}\rho_{veg} & \text{if} & \left[\left(0.1h_{tree}-D_{canopy}\right)\geq W_{stream}\right] \end{array}\right.$$

where *ρ_veg_*is the average density of the vegetation canopy, which ranges from 0 to 1 (unitless) and should be determined by the user. The effective shading width is computed using Beer's Law as [11]

(20) $$W_{eff}=\left\{ \begin{array}{ccc} \left(W_{shade}-D_{i}-W_{overhang}\right)\left(1-e^{-\lambda L_{avg}}\right) & \text{if} & SA_{veg}>SA_{i=1or3}\\ \left(W_{shade}-D_{i}-W_{overhang}\right) & \text{if} & SA_{i=1or3}>SA_{veg} \end{array}\right.$$

where *λ*, the radiation extinction coefficient, is calculated as a function of the leaf area index, *LAI* from the Eq. (2) of [14] and *L_avg_* is the average path length of direct solar radiation through the shaded zone around the river (m) [13]. When canopy overhangs the river surface, the model uses an adjusted effective width $W_{eff}^{\prime }$ computed as

(21) $$W_{eff}^{\prime }=W_{eff}+W_{overhang}$$

Using the adjusted direct radiation affected by topographic shading ($\Phi_{shortwave}^{{}^{\prime }direct}$) and the calculated adjusted effective width, the net direct solar radiation affected by the topographic and shading barriers reaching to the surface,$\Phi_{shortwave}^{{}^{\prime \prime }direct}$ can be calculated as shown in Eq. (14) [13].

### Latent heat flux

The latent heat flux in Eq. (5) is a negative upward flux representing evaporative cooling [10,15]. The latent heat flux is computed as [2]

(22) $$\Phi_{latent}=-\rho L_{e}E$$

where *L_e_* is the latent heat of vaporization (J/kg), and *E* is the evaporation rate (m/s). The i-Tree Cool River Model provides two methods for calculation of *E* from open water, the Penman-Monteith combination method using Eq. (30) of [10], and a mass transfer method using Eq. (2-96) of [2].

### Sensible heat flux

Sensible convection of heat in Eq. (5) represents the heat exchange between the surface of the water and the air [15]. The i-Tree Cool River Model provides three flexible methods to calculate the sensible heat flux, first and second methods (Eqs. (23) and 24) based on the Bowen ratio of sensible to latent heat, and the third method (Eq. (25)) based on the sensible heat. The simpler of the two Bowen ratio methods is based on [2]

(23) $$\Phi_{sensible}=B_{r}\Phi_{latent}$$

where *Br* is the Bowen ratio. The more complex of the Bowen ratio methods is based on [16]

(24) $$\Phi_{sensible}=B_{r}\rho_{w}\gamma NU_{wind}\left(T_{air}-T_{w}\right)$$

where *γ* is the latent heat of vaporization (2.4995 × 10^6^ J/kg), *N* is an empirical constant (1.59 × 10^−9^ s/m.mb) and *U_wind_* is wind speed (m/s). The sensible-heat-based method considers wind speed as a driver of the convective flux, based on [9], given by [2] as

(25) $$\Phi_{sensible}=-K_{H}U_{wind}\left(T_{w}-T_{air}\right)$$

where *K_H_* is the heat exchange coefficient for sensible heat (J/ m^3 o^C).

### Bed sediment heat flux

The bed sediment heat flux in Eq. (5) represents the heat conduction between the bed sediment and the water column in the cross-section and is rate limited by the size and conductance properties of the substrate. The approach used in the i-Tree Cool River model modifies Eq. (2-90) of [2] as

(26) $$\Phi_{se\dim ent}=2K_{CL}\frac{T_{bed}-T_{w}}{\frac{d_{w}}{2}}$$

where *K_CL_* is the volumetric weighted thermal conductivity (J/ms °C), *T_bed_* is the bed temperature (°C), and *d_w_* is the average river depth in the cross-section (m). The sediment interface with the river water is the *T_bed_* in Eq. (26); some applications prescribe *T_bed_* to a depth below the interface. The mid-depth of the river, *d_W_/2,* is used in Eq. (26) to represent a mid-point of the river water temperature reservoir. By solving for the heat fluxes of Eq. (5), the i-Tree Cool River model can solve Eq. (2) and provide the heat flux reaction term, *R_e_*, for the governing advection-dispersion-reaction Eq. (1) used to simulate river temperatures.

## Coupling the i-Tree cool river model with the HEC-RAS

For coupling the i-Tree Cool River model [3] with the HEC-RAS [17], Eq. (1) was updated to include HEC-RAS model river water surface materials as [19]

(27) $$T=T^{t-1}+\Delta t\left[V\frac{T_{up}-T}{\Delta x}-D_{L}\frac{T_{up}-2T-T_{dn}}{{\left(\Delta x\right)}^{2}}\right]+R_{h}+R_{i}$$

where similar to Eq. (1), *T* (°C) is the temperature at a river cross-section, the superscript *t-1* indicates the prior time, *∆t* (s) is the time step, the subscript *up* indicates the upstream cross-section, the subscript *dn* indicates the downstream cross-section, *V* (m/s) is the cross-section velocity, *∆x* (m) is the length of the reach segment bounded by the cross-section and upstream cross-section, *D_L_* (m^2^/s) is the reach longitudinal dispersion computed as a function of cross-section velocity and depth, *R_h_* (°C) is the loss or gain (i.e., reaction term) of temperature due to heat flux, and *R_i_* (°C) is the loss or gain of temperature due to lateral inflows [3,17]. The velocity is determined by a separate set of hydraulic equations, which in steady-state mode involves simultaneously estimating depth and velocity to satisfy the conservation of energy, mass, and momentum, i.e., the 5-step algorithm used in HEC-RAS [17], which is an alternative to the Newton-Raphson algorithm used in i-Tree Cool River [18,19].

To combine the i-Tree Cool River with the HEC-RAS model simulation of water surface profiles, Eq. (27) was modified to include the velocity and discharge data from the HEC-RAS model. To that end, the retention time was added to the equation in order to apply the HEC-RAS model calculated velocity data [18,19].

(28) $$T=\frac{\left[T_{up}^{t-1}+\Delta t_{r}R_{h2}\right]Q}{Q}+\frac{R_{i2}}{Q}$$

where $T_{up}^{t-1}$ is the temperature of the upstream cross-section at the prior time step, *∆t_r_* (s) is the retention time step defined as $\Delta t_{r}=\Delta x/V$and *V* (m/s) is the HEC-RAS velocity at the cross-section, *Q* (m^3^/s) is the HEC-RAS discharge at the cross-section, and *R_h_*_2_ is the heat flux reaction term, and *R_i2_* is lateral inflows reaction term. An updated heat flux reaction term was considered for Eq. (28) [18,19],

(29) $$R_{h2}=\left[\frac{\phi }{\rho \cdot C_{p}\cdot y}-D_{L}\frac{\left(\frac{\phi }{\rho \cdot C_{p}\cdot y\cdot V}-\frac{\phi_{up}}{\rho \cdot C_{p}\cdot y_{up}\cdot V_{up}}\right)}{\Delta x}\right]$$

where *φ* is defined to include the i-Tree Cool River model tree-based terms (e.g. Eq. (5)). The lateral inflows reaction term equation also was updated as [18,19],

(30) $$R_{i2}=\frac{Q_{a}T_{a}+Q_{b}T_{b}+Q_{c}T_{c}}{Q_{a}+Q_{b}+Q_{c}}$$

where subscripts a, b, and c are defined as the i-Tree Cool River form with Eq. (3) above.

Considering this set of the formulation, we coupled the i-Tree Cool River model with the HEC-RAS model in order to utilize water surface profile data from the HEC-RAS model and thereby coordinate evaluations of thermal and flood management impacts. By coupling the hydraulic transport model of HEC-RAS with the temperature transport model of i-Tree Cool River, Eq. (28) can lead to differences in the volume of water predicted by the two transport models. This difference is due to the first right-hand side term in Eq. (28) representing temperature transport as plug flow, with upstream temperature from the prior time step replacing downstream temperature in the current time step, even when upstream and downstream volumes may not be equal for each reach segment. To avoid this continuity error, the i-Tree Cool River model maintains the HEC-RAS model volume for each reach segment and uses a cross-section spacing of 5 m or less to constrain the error in the temperature estimate [18,19].

## Model inputs

The i-Tree Cool River Model uses a set of input data including upstream boundary condition, steady or unsteady discharge hydrograph for the river and lateral storm sewer inflows, groundwater and hyporheic exchange data, streambed temperature, meteorological data, etc. for the simulation process, which can be imported to the model using the DAT files (the input data for temperature modeling in time and space) and the XML file (which includes the initial necessary data for the beginning of the simulation). The i-Tree Cool River model has a function to linearly interpolate the input data based on the defined ∆x and ∆t and the input files can be imported at different intervals. The name of the required input files and a brief description for each input file represented in Table 1. Input files contain three TXT files including the impervious cover, land cover, and tree cover which are converted from raster to ASCⅡ and are not effective in the simulation process of the i-Tree Cool River model. Table 2 presents the alternative methods for obtaining the input data for the i-Tree Cool River model input data. Table 3 also represents the description of the initial parameters which are imported to the model in the XML file. More information such as sample inputs and outputs as well as a detailed manual can be downloaded at http://www.itreetools.org/research_suite/coolriver/.

**Table 1:** List of the input files required for the simulation process of the i-Tree Cool River Model.

Input file	The parameter name	Description
BedData.dat	Number	The number of observations indicates the locations of the observed streambed data.
	Distance (m)	Distances through the river reach where the streambed observations are recorded.
	Depth of Measurement (m)	Depth at which groundwater temperatures are recorded in each cross-section
	GW_Temp (°C)	Groundwater temperature in the downstream.
	Type	Bed-sediment type which can be clay, silt, sand, or gravel.
	Horizontal Bed Conductivity (mm/s)	Horizontal effective thermal conductivity in each observed cross-section.
	Bed Particle Size (mm)	Bed particle size (Bedient and Huber, 1992, Rosgen, 1996) in the observed location.
	Embeddedness (fraction)	Embeddedness in each considered cross-section.
DEM.txt	Elevation data for calculating slope and aspect for calculating the hillslope effect on energy flux which can be converted from raster file to ASCІІ in Arc Map. The raw DEM data can be downloaded from the National Map Viewer.	
Discharge.dat	Number	The number of observations indicates the locations of the observed groundwater data.
	Distance (m)	Distances through the river reach where the magnitude of groundwater flow is recorded
	Q_GW (cms)	Groundwater discharge.
Inflow.dat*	Number	The number of observations which indicates the number of the time steps for the hydrographs of the river and lateral inflows.
	Inflow Rate Storm (cms)	Discharge rates of the river in upstream at each timestep defining the hydrograph in steady or unsteady mode.
	Inflow Temp Storm (°C)	Observed stream temperatures corresponding to the river hydrograph timesteps in upstream.
	Inflow Rate 1 (cms)	Discharge rates of the lateral storm sewer inflow at each timestep for the first location defining the hydrograph in steady or unsteady mode.
	Inflow Temp 1 (°C)	Observed stream temperatures corresponding to the first lateral storm sewer inflow hydrograph timesteps.
	Inflow Rate 2 (cms)	Discharge rates of the lateral storm sewer inflow at each timestep for the second location defining the hydrograph in steady or unsteady mode.
	Inflow Temp 2 (°C)**	Observed stream temperatures corresponding to the second lateral storm sewer inflow hydrograph timesteps.
	* The First row of the input file below the headings should be considered as the location of each hydrograph. The river's hydrograph gets 1 m indicating the upstream and other lateral inflows receive their own location from the upstream. ** The number of lateral inflows can be changed in the code by the user.	
Morphology.dat	Number	The number of observations indicates the locations of the measured geomorphic data.
	Distance (m)	Distances through the river reach corresponding with the cross-sections where the geomorphic data are recorded.
	Area (m^2^)	The initial cross-sectional wetted area of the river channel; dummy variable.
	Width (m)	Channel bottom width.
	Depth (m)	Initial channel water depth.
	Discharge (cms)	River discharge magnitude at the location where the geometric data are measured.
	Slope	Channel longitudinal slope
	Row#**	The row number in the DEM file where the cross-section is located.
	Column#**	The column number in the DEM file where the cross-section is located.
	Longitude (deg)**	Longitude of the cross-section in the geographic coordinate system.
	Latitude (deg)**	Latitude of the cross-section in the geographic coordinate system.
	Z (H:V)	The side slope of the trapezoidal channel, equivalent to horizontal distance to vertical distance ratio. Same value for both channel sides.
	** These input data are required for calculating the slope and aspect of each cell to apply the values on the hillslope effect and the shortwave radiation. In case of using fixed magnitudes for the shade factor and view-to-sky values, these values are not effective in the simulation process.	
Shading.dat*	Number	The number of observations reflecting the locations of the measured shading information.
	Distance (m)	Distances through the river reach corresponding with the cross-sections where the shading information is recorded.
	EastBankH (m)	The height of the bankfull^a^ at the measured cross-section on the Eastside.
	EastTreeH (m)	The height of the canopy at the measured cross-section on the Eastside.
	EastBuildingH (m)	The height of the building at the measured cross-section on the Eastside.
	EastBankDist (m)	Distance from the bankfull to the edge of the water at the measured cross-section on the Eastside.
	EastCanDist (m)	Distance from the canopy to the edge of the water at the measured cross-section on the Eastside.
	EastBuildingDist (m)	Distance from the building to edge of the water at the measured cross-section on the Eastside.
	EastBufferW (m)	The magnitude of the canopy buffer at the location of the measured cross-section on the Westside
	WestBankH (m)	The height of the bankfull at the measured cross-section in the Westside.
	WestTreeH (m)	The height of the canopy at the measured cross-section on the Westside.
	WestBuildingH (m)	The height of the building at the measured cross-section on the Westside.
	WestBankDist (m)	Distance from the bankfull to the edge of the water at the measured cross-section on the Westside.
	WestCanDist (m)	Distance from the canopy to the edge of the water at the measured cross-section on the Westside.
	WestBuildingDist (m)	Distance from the building to edge of the water at the measured cross-section on the Westside.
	WestBufferW (m)	The magnitude of the canopy buffer at the location of the measured cross-section on the Westside
	Elevation (m)	The elevation of the cross-section.
	StreamAzimuth (deg)	The stream azimuth at the location of the measured cross-section.
	* These input data are required for calculating the topographic, canopy (tree), and building shade angle and view-to-sky factor to apply the values to the hillslope effect and the shortwave radiation. In case of using fixed magnitudes for the shading factor and view-to-sky values, these values are not effective in the simulation process.	
ShadingPercent.dat*	Number	The number of observations reflecting the locations of the shading factors.
	Distance (m)	Distances through the river reach corresponding with the cross-sections where the shading factor and the view-to-sky values are calculated.
	ShadeFactor	The value of cross-section shade factor for daily average, with 0 for no shading, and 1 for full shading. This is representative of the entire channel, and can be the average for right and left banks. It can be estimated using site visits, aerial photos, or best estimates.
	View-to-Sky	The value of View-to-Sky in the desired cross-section which is 1-ShadeFactor
	* In case the topographic, canopy, and building heights and distances are considered for shading calculations, the magnitude of ShadeFactor and View-to-Sky are not effective in the simulation process.	
SolarRadiation.dat* The number of entries in this file should match the attribute value of totTime in the config file (see Table 2)	yyyymmdd	The date of the simulation period.
	Hr: Min: Sec	The time of the simulation period.
	DirSW (W/m^2^)	Direct shortwave radiation at the edge of the atmosphere.
	DiffSW (W/m^2^)	Diffuse shortwave radiation at the edge of the atmosphere.
	* Source: National Solar Radiation Database (NSRDB)	
Time.dat	Number	The number of time steps.
	Time (s)	The desired time step for the output intervals.
Weather.dat* The number of entries in this file should match the attribute value of totTime in the config file (see Table 2)	yyyymmdd	The date of the simulation period.
	Hr: Min: Sec	The time of the simulation period.
	Tair (F)	Air temperature.
	WndSpd (m/s)	Wind speed.
	Precip (m/h)	Precipitation rate.
	Cloudiness	The magnitude of the cloudiness.
	Humidity	Relative humidity.
	obsT_x0 ( °C)	Observed river temperature in the upstream.
	sedT ( °C)	Riverbed temperature.
	* National Center for Environmental Information	

a The water level, or stage, at which a stream, river or lake is at the top of its banks and any further rise would result in water moving into the flood plain.

**Table 2:** The alternative methods for obtaining the input data for the i-Tree Cool River model input data.

Input file	Parameter	Explanation / Obtaining method	Alternative Obtaining method/website
BedData.dat	Depth of Measurement (m)	Typically set to 2 m below the bed for measurement of groundwater temperature.	
	GW_Temp (°C)	At 2 m depth is estimated as average annual air temperature, possibly with seasonal adjustment.	NOAA's NCDC database: https://gis.ncdc.noaa.gov/maps/ncei/ or National Solar Radiation Database: https://rredc.nrel.gov/solar/old_data/nsrdb/
	Type	Bed substrate material type is based on the estimate of site visit and is selected from a range of 4 types within model code: cobble, gravel, sand, clay	
	Horizontal Bed Conductivity (mm/s)	Values are associated with bed substrate material type and can be set based on hydrology reference materials or site testing. Boyd and Kasper (2003) has values for these.	
	Bed Particle Size (mm)	Values are associated with bed substrate material type and can be set based on hydrology reference materials or site testing. Boyd and Kasper (2003) has values for these.	
	Embeddedness (fraction)	Values are associated with bed substrate material type and can be set based on morphology reference materials or site testing. Boyd and Kasper (2003) has values for these.	
DEM.txt		Extent of DEM can extend out to entire watershed or be limited to the river corridor, and used tp compute hillslope effect on energy flux.	National Map Viewer: https://viewer.nationalmap.gov/advanced-viewer/Google Engine: https://earthengine.google.com/
Discharge.dat	Q_GW (cms)	Estimated as the difference in discharge at the downstream and upstream section of the reach. The discharge at each end of the reach could be from i-Tree Hydro simulations, observation, or USGS StreamStats.	USGS's Stream stats: https://streamstats.usgs.gov/ss/
Inflow.dat	Inflow Rate (cms)	Lateral flow from steady or unsteady state, and is either estimated or observed. Estimates could come from i-Tree Hydro model simulation.	SWMM: https://www.epa.gov/water-research/storm-water-management-model-swmm
	Inflow Temp (°C)	Estimate or observation, for steady or unsteady state. Anticipate a new algorithm could be added to Unified Hydro (w/ Cool Air) to simulate this value.	A non-linear regression equation based on air temperature: Mohseni et al., (1998) https://agupubs.onlinelibrary.wiley.com/doi/abs/10.1029/98WR01877
Morphology.dat used for 3 model options: Crank Nicholson, Runge Cutta, or Explicit Finite Difference. Not used in model option: HEC-RAS. Alternatively use: HecRasData.dat, which contains: downstream distance (between cross-sections); discharge; minimum channel elevation; water surface elevation; velocity; area; top width; water surface slope; wetted perimeter. All of these outputs are generated by HEC-RAS as SI units.	Distance (m)	Distances to downstream channel cross-sections. Estimated based on map or site analysis such as NHD, or based on systematic intervals.	National Hydrography Data: https://www.usgs.gov/core-science-systems/ngp/national-hydrography National Map Viewer: https://viewer.nationalmap.gov/advanced-viewer/ Google Engine: https://earthengine.google.com/
	Area (m^2^)	Initial value of the cross-sectional area, dummy variable not used by the model.	
	Width (m)	Cross-sectional bottom width for trapezoidal channel (0 value for triangular). Could be estimated: a) using field surveys; or b) using a modification of hydraulic geometry relations to get channel geometry at bankfull based on the watershed area, which is provided by some USGS SteamStats sites and NHD - scale values for lower flow.	
	Depth (m)	Initial time step of water depth. Could be estimated: a) using field surveys; or b) using a modification of hydraulic geometry relations to get channel geometry at bankfull based on the watershed area, which is provided by some USGS SteamStats sites and NHD - scale values for lower flow.	
	Discharge (cms)	Upstream boundary flow from steady or unsteady state and is either estimated or observed. Estimates could come from i-Tree Hydro model simulation.	
	Slope	Longitudinal bed slope. Could be estimated: a) using field surveys; b) using DEM analysis, or c) USGS SteamStats sites and NHD.	USGS's Stream stats: https://streamstats.usgs.gov/ss/
	Row#	Associated with the DEM.txt input, and is input row number (0 is the first row) of each river cross-section. Used to compute slope and aspect for radiation estimates. Could estimate from observation or overlaying NHD image of the river on DEM in ArcGIS.	National Map Viewer: https://viewer.nationalmap.gov/advanced-viewer/ Google Engine: https://earthengine.google.com/
	Column#	Associated with the DEM.txt input, and is input column number (0 is the first column) of each river cross-section. Used to compute slope and aspect for radiation estimates. Could estimate from observation or overlaying NHD image of the river on DEM in ArcGIS.	National Map Viewer: https://viewer.nationalmap.gov/advanced-viewer/ Google Engine: https://earthengine.google.com/
	Longitude (deg)	Associated with each river cross-section. It may not vary with short river. Could estimate from observation or map analysis.	National Map Viewer: https://viewer.nationalmap.gov/advanced-viewer/ Google Engine: https://earthengine.google.com/
	Latitude (deg)	Associated with each river cross-section. It may not vary with short river. Could estimate from observation or map analysis.	National Map Viewer: https://viewer.nationalmap.gov/advanced-viewer/ Google Engine: https://earthengine.google.com/
	Z (H:V)	Channel side slope for trapezoidal channel banks, the same value used on each bank. Could estimate from field observation, design decision, or image analysis.	National Map Viewer: https://viewer.nationalmap.gov/advanced-viewer/ Google Engine: https://earthengine.google.com/
Shading.dat	All the parameters	All the parameters for this input data (2nd method of calculating the longwave and shortwave radiations) are based on direct measurements at the field sites or of maps and images.	Lidar surveying data (if it is available) https://viewer.nationalmap.gov/basic/ https://gisgeography.com/top-6-free-lidar-data-sources/
ShadingPercent.dat	ShadeFactor	The value of cross-section shade factor for daily average, with 0 for no shading, and 1 for full shading. This is representative of the entire channel, and can be the average for right and left banks. It can be estimated using: a) site visits; b) aerial photos with the manual interpretation or TTools module in Heat Source Model; or c) scenario estimates.	The heat source model's TTools module: https://www.oregon.gov/deq/wq/tmdls/Pages/TMDLs-Tools.aspx
	View-to-Sky	The value of View-to-Sky in the desired cross-section which is 1-ShadeFactor	
SolarRadiation.dat	Direct and Diffuse shortwave radiation data	National Solar Radiation Database: https://rredc.nrel.gov/solar/old_data/nsrdb/	i-Tree Hydro preprocessor:
Time.dat	Time (s)	The desired time step for the output data intervals.	
Weather.dat	Tair (F)	Weather input data of air temperature	NOAA's NCDC database: https://gis.ncdc.noaa.gov/maps/ncei/ National Solar Radiation Database: https://rredc.nrel.gov/solar/old_data/nsrdb/
	WndSpd (m/s)	Weather input data of wind speed	
	Precip (m/h)	Weather input data of precipitation	
	Cloudiness	Weather input data of cloudiness	
	Humidity	Weather input data of humidity	
	obsT_x0 (°C)	Upstream boundary river temperature, for each time step. Estimated with observation or non-linear regression with air temperature.	A non-linear regression equation using air temperature: Mohseni et al., (1998) https://agupubs.onlinelibrary.wiley.com/doi/abs/10.1029/98WR01877
	sedT (°C)	Channel bed substrate temperature, for each time step. Estimated with observation or model prediction based on solar radiation or temperature data.	NOAA's NCDC database: https://gis.ncdc.noaa.gov/maps/ncei/ National Solar Radiation Database: https://rredc.nrel.gov/solar/old_data/nsrdb/

**Table 3:** List of the parameters which should be specified in the XML file for the simulation process of the i-Tree Cool River Model.

Key tags	The parameter name	Description
Inputs	Input_Folder	The address of the input folder directory that could be different for every machine.
Outputs	Output_Folder	The address of the output folder directory that could be different for every machine.
Temporal_Domain	starttime	The date of the starting of the simulation in the yyyymmdd format.
	endtime	The date of the finishing of the simulation in the yyyymmdd format.
	Timeinterval (h)	The intervals of the timesteps in the simulation process.
	startingHour	The starting hour of the simulation process.
	SWMethod	The code of the desired method for calculating the shortwave and longwave radiation energy fluxes: • Number 1 is for solving shortwave and longwave radiation fluxes based on the sky-to-view factor shading angles calculated from the provided heights and distances of the hillslope, vegetation, and building. • Number 2 is for solving shortwave and longwave radiation fluxes based on the fixed values for sky-to-view and shading factors.
	initialTemp	The observed river temperature of the upstream cross-section at the first timestep.
	canDensity	The density of the vegetation in the study area.
	roughness	Manning's roughness coefficient (n).
	LAI	The magnitude of the leaf area index in the study area.
Shading_inputs	tarLat	Representative latitude of the study area.
	tarLong	Representative longitude of the study area.
	tarRow	The row number of the representative cell of the study area.
	tarCol	The column number of the representative cell of the study area.
	standardMeridian	The meridian ranking of the time zone in the study area.
	totDist	The total distance of the river.
	calcMethod	The desired method to calculate the river temperature 1: Solution Method=Crank-Nicolson 2: Solution Method=Explicit finite difference 3: Solution Method=HEC-RAS based method 4: Solution Method=Rung-Kutta
	depthOfBed	The measured or assumed depth of the river bed
	totTime	A total number of the simulation process in an hour.
	sensMethod	The code of the desired method for calculating the sensible heat flux: 1: The method as a function of the wind speed (See Eq. S14, Abdi & Endreny 2019) 2: The method as a function of Bowen ration and latent heat (See Eq. S12, Abdi & Endreny 2019) 3: The method as a function of Bowen ratio, latent heat, and an empirical constant described in Sun et al., (2015) (See Eq. S13, Abdi & Endreny 2019)
	Sediment_method	The method for calculating the sediment flux 1: Solving sediment flux based on RBM model 2: Solving sediment flux based on Heat Source model (for HEC-RAS based method)
Unsteady_inputs	dx	The considered distance (Δx) for the numerical finite difference method.
	dt	The considered timestep (Δt) for the numerical finite difference method.

## Model outputs

After the simulation process, the i-Tree Cool River model generates four comma-separated-value (CSV) files for the predicted temperatures and the net value of the heat flux in minute and hour based intervals (Table 4). The CSV files are two-dimensional vectors in which each row shows each meter of the river reach and each column represents each timestep of the running process. The user can add extra output files in the code as well.

**Table 4:** List of the default outputs created by simulating the i-Tree Cool River Model.

Output filename	Description
2D_Hourly_Temperature.csv	A two-dimensional (2D) matrix including the hourly simulated river temperature. The columns reflect the timestep (∆T) and the rows show the intervals (∆X).
2D_Hourly_TotalFlux	A two-dimensional (2D) matrix including the hourly simulated total flux as a combination of the longwave, shortwave, latent, sensible, and sediment fluxes. The columns reflect the timestep (∆T) and the rows show the intervals (∆X).
2D_Minutely_Temperature	A two-dimensional (2D) matrix including the simulated river temperature. The timestep in this output file is one minute and the spatial intervals are one meter. The columns reflect the timestep (∆T) and the rows show the intervals (∆X).
2D_Minutely_TotalFlux.csv	A two-dimensional (2D) matrix including the simulated total flux as a combination of the longwave, shortwave, latent, sensible, and sediment fluxes. The timestep in this output file is one minute and the spatial intervals are one meter. The columns reflect the timestep (∆T) and the rows show the intervals (∆X).

## Running the i-Tree cool river m

We provide an application file (iTreeCoolRiver.exe) in the iTreeCoolRiver_x64/86exe folder (can be downloaded at http://www.itreetools.org/research_suite/coolriver/). The model executable is called at the command line along with an extensible markup language (XML) file, which includes the required initial information in the sample input files folder. The target platform for this application is Windows 10. In case of any trouble running this application for a different platform, the users should consider creating a build for their platform (see Compile and Build iTreeCoolRiver via Visual Studio 2017 section). The easiest way to get the application “up and running” is to download the compressed iTreeCoolRiver.exe file (the iTreeCoolRiver_x64/86exe folder) and extract all to the C: drive. (If the user chooses a different location, it will be necessary to edit the Inputs and Outputs tag of the iTreeCoolRiver.xml config file accordingly and specify the correct config location when running the application. See the Input Data section, Table 2).

**Fig. 2:**
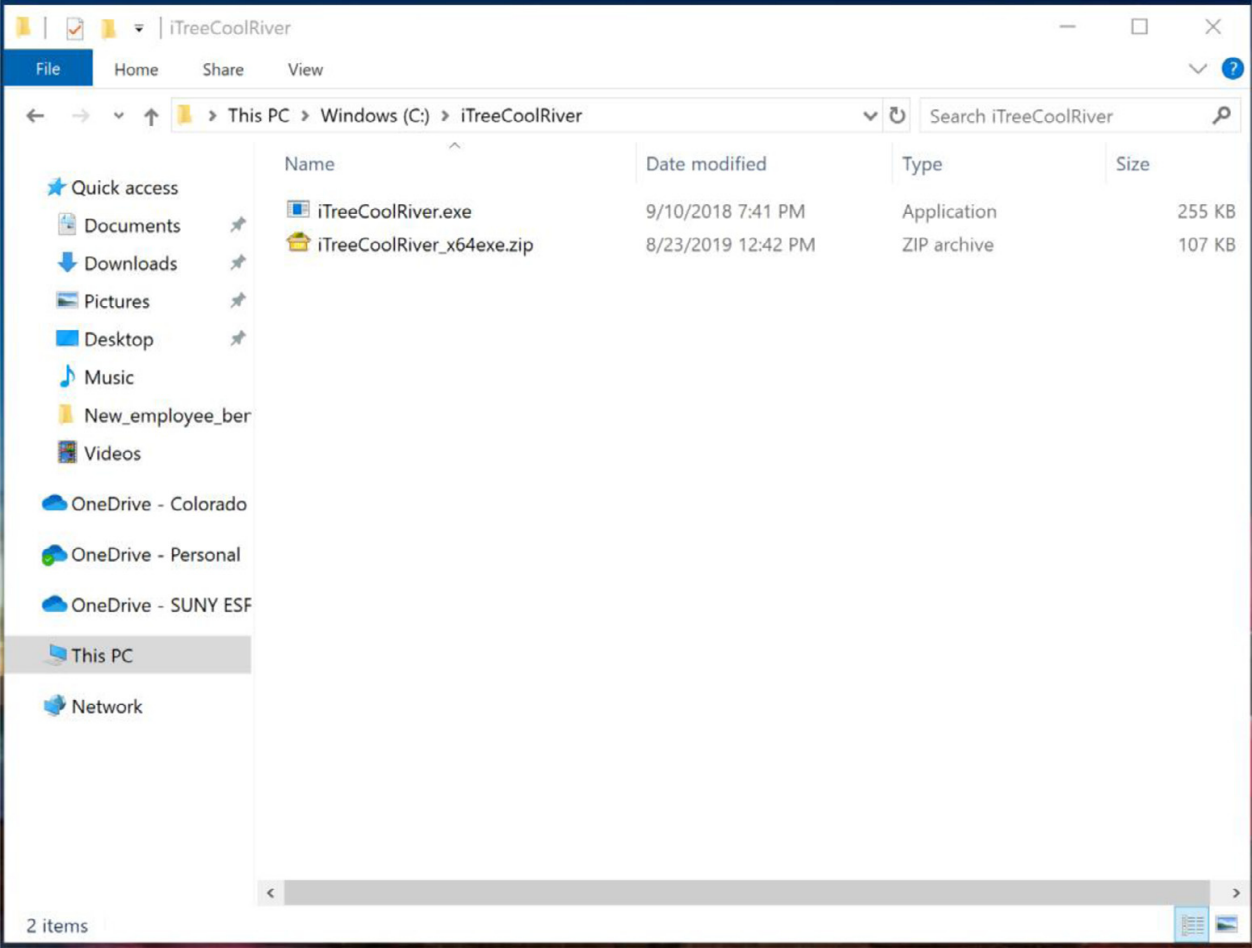
Uncompressing the “iTreeCoolRiver_x64exe.zip” file to get access to the executable file.
• Uncompress the “iTreeCoolRiver_SampleData.zip” file to “c:\iTreeCoolRiver”, creating “C:\iTreeCoolRiver\ExampleInputs” and C:\iTreeCoolRiver\ ExampleOutputs (Fig. 3)

The required steps for running the i-Tree Cool River model:

• Uncompress the “iTreeCoolRiver_x64exe.zip” file to “c:\iTreeCoolRiver (Fig. 2)

**Fig. 3:**
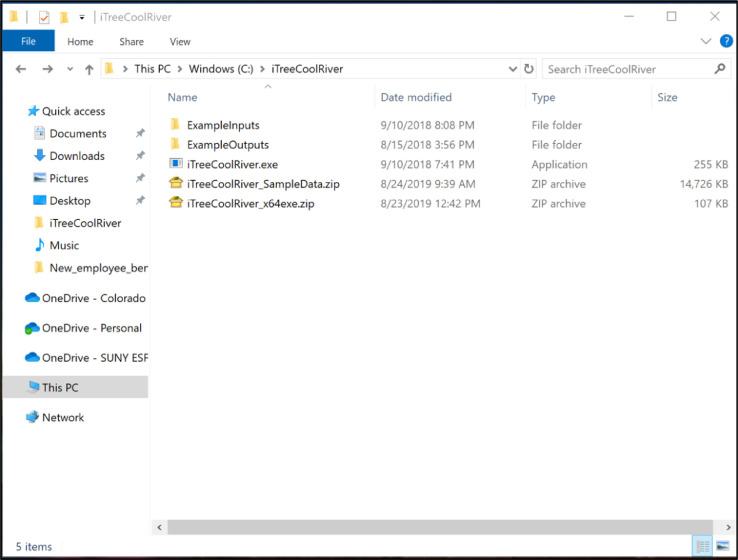
Uncompressing the “iTreeCoolRiver_SampleData.zip” file to get the sample input and output folders.
• To run the model, either
  ○ In a DOS Command Prompt navigate to “C:\ iTreeCoolRiver\” and type the name of the executable and the config file and its path: “C:\ iTreeCoolRiver> iTreeCoolRiver.exe C:\ iTreeCoolRiver\iTreeCoolRiver.xml” (Fig. 4)

**Fig. 4:**
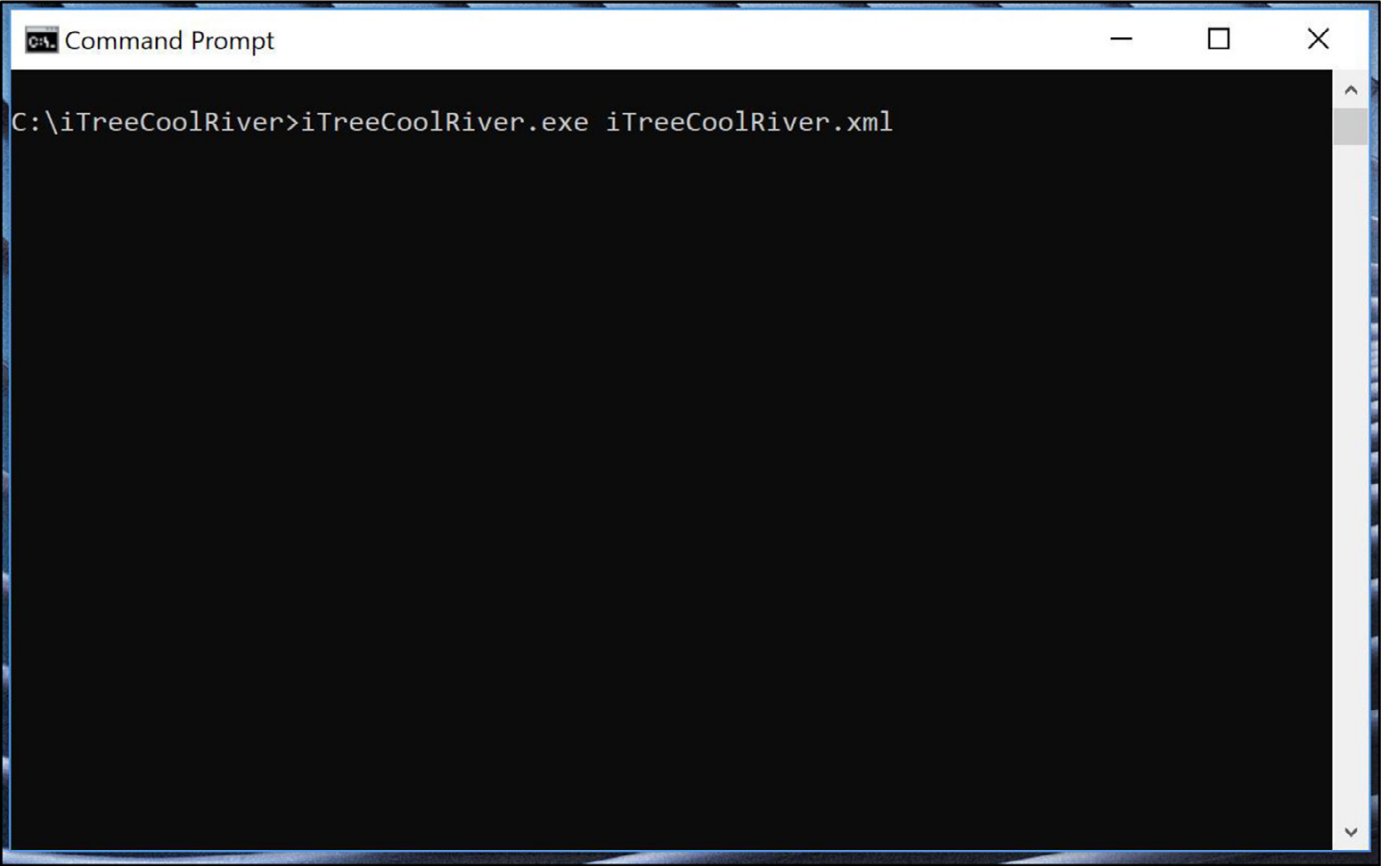
Executing the i-Tree Cool River model in a DOS Command Prompt.
  ○ In a Windows Explorer double click on the iTreeCoolRiver.exe file and a DOS Command Prompt will open, type the config file and path:

“C:\ iTreeCoolRiver\iTreeCoolRiver.xml (Fig. 5)

**Fig. 5:**
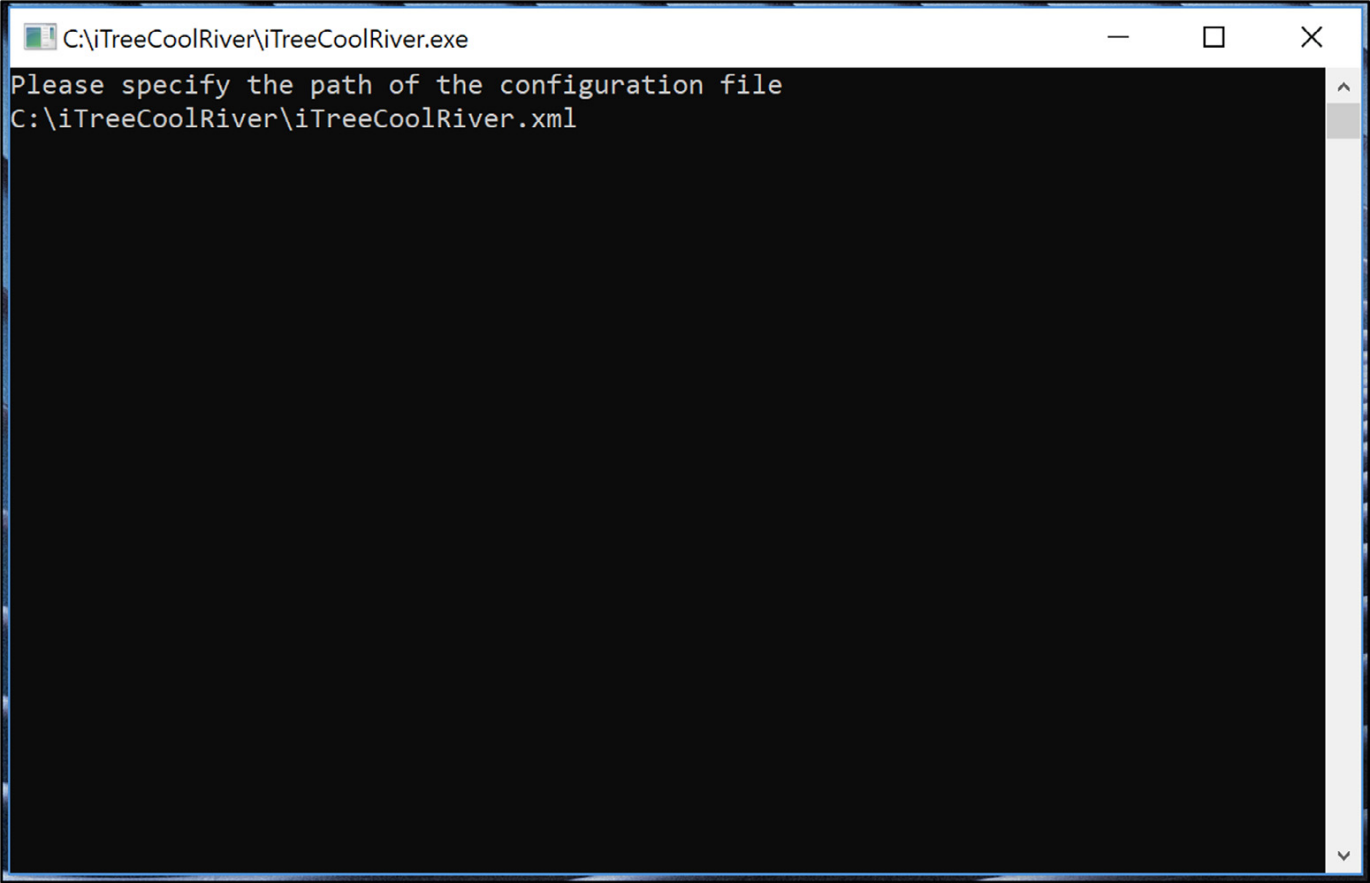
Executing the i-Tree Cool River model in a Windows Explorer.

**Fig. 6:**
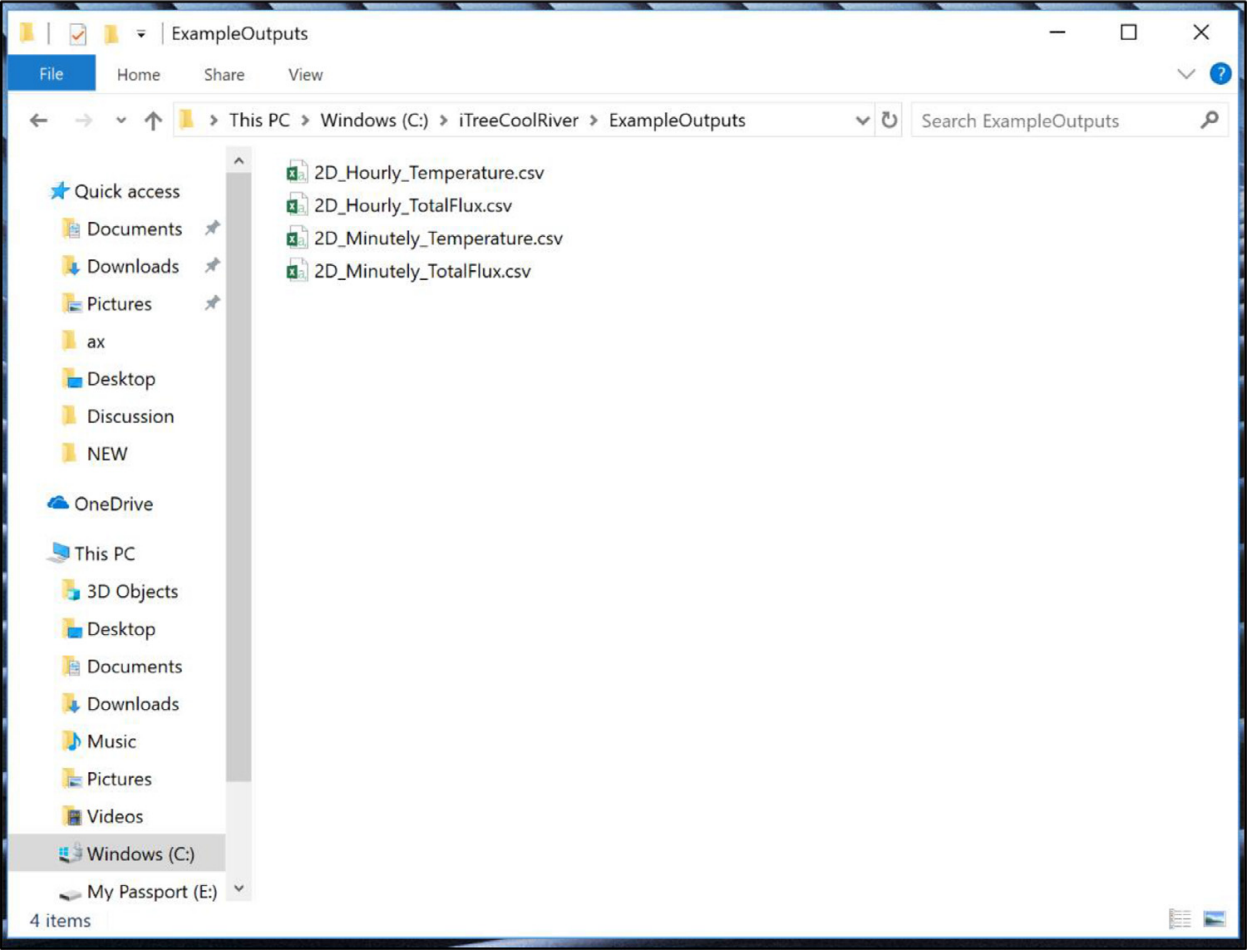
Outputs after executing the sample input data (see the link http://www.itreetools.org/research_suite/coolriver/).

• You will find the output of this run in the ExampleOutputs folder (Fig. 6).

The detailed steps for compiling the building the i-Tree Cool River model solution file in Visual Studio 2017 also are described in the model's manual which can be downloaded at http://www.itreetools.org/research_suite/coolriver/.

## Method validation

Using the model described in the previous sections, we simulated river water temperature for several rivers in different climates for the steady and unsteady states. We used the i-Tree Cool River model in order to simulate the effects of the riparian vegetation and lateral inflows from different sources including urban storm sewers, hyporheic exchange inflows, and groundwater on river temperature in the unsteady state. The model was tested along 1500 m of a New York mountain river with riparian forest and urban areas during 30 h with two summer storm events in 2007 [3,20]. The model simulated hourly river temperature for 30 h simulation period with a 2-hour rain event in June 11 and 12, 2007 with an R^2^ of 0.98. Our simulations showed that the river water temperatures are sensitive to the inflows of storm sewers, subsurface inflows, riparian shading, and upstream boundary condition temperatures for both steady and unsteady conditions. We removed the riparian shading from the simulation and the R^2^ decreased 0.88, indicating the importance of riparian shading in river thermal modeling. We also removed the stormwater inflows from the simulation and observed that the R^2^ decreased from 0.98 to 0.92, and by removing the subsurface inflows, we saw that the R^2^ decreased to 0.94 [3,20].

We updated our model to couple it with the HEC-RAS as described in section 2 and tested our updates study updates by simulating the impacts of warm surface runoff, lack of riparian forests, and impervious channels that transfer heat and block cool subsurface flows on river water temperature [18] to address the urban river syndrome [21]. We tested the updated i-Tree Cool River model on a 2 km mountain river within the New York City drinking water supply area (Sawmill, SM, Creek in Tannersville, NY), and used for base case and restoration scenarios on the 17.5 km reach of the Los Angeles (LA) River in a 48 h simulation period. The model simulated the LA River average temperature in the base case decreased from 29.5 °C by 0.3 °C when warm surface inflows were converted to cooler groundwater inflows by terrestrial green infrastructure; by 0.7 °C when subsurface hyporheic exchange was increased by removal of armoring and installation of riffle-pool bedforms; by 3.6 °C when riparian forests shaded the river; and by 6.4 °C when floodplain forests were added to riparian forests to cool surface reservoirs and local air temperatures [18,22]. We observed that by applying all four restoration treatments on the model, the average river temperature decreased by 7.2 °C [18,22].

## Declaration of Competing Interests

The authors declare that they have no known competing financial interests or personal relationships that could have appeared to influence the work reported in this paper.
